# Designs for the simultaneous inference of concentration–response curves

**DOI:** 10.1186/s12859-023-05526-3

**Published:** 2023-10-19

**Authors:** Leonie Schürmeyer, Kirsten Schorning, Jörg Rahnenführer

**Affiliations:** Department of Statistics, TU Dortmund University, Dortmund, Germany

**Keywords:** Optimal design, Gene expression, Nonlinear regression, High-dimensional data

## Abstract

**Background:**

An important problem in toxicology in the context of gene expression data is the simultaneous inference of a large number of concentration–response relationships. The quality of the inference substantially depends on the choice of design of the experiments, in particular, on the set of different concentrations, at which observations are taken for the different genes under consideration. As this set has to be the same for all genes, the efficient planning of such experiments is very challenging. We address this problem by determining efficient designs for the simultaneous inference of a large number of concentration–response models. For that purpose, we both construct a *D*-optimality criterion for simultaneous inference and a *K*-means procedure which clusters the support points of the locally *D*-optimal designs of the individual models.

**Results:**

We show that a planning of experiments that addresses the simultaneous inference of a large number of concentration–response relationships yields a substantially more accurate statistical analysis. In particular, we compare the performance of the constructed designs to the ones of other commonly used designs in terms of *D*-efficiencies and in terms of the quality of the resulting model fits using a real data example dealing with valproic acid. For the quality comparison we perform an extensive simulation study.

**Conclusions:**

The design maximizing the *D*-optimality criterion for simultaneous inference improves the inference of the different concentration–response relationships substantially. The design based on the *K*-means procedure also performs well, whereas a log-equidistant design, which was also included in the analysis, performs poorly in terms of the quality of the simultaneous inference. Based on our findings, the *D*-optimal design for simultaneous inference should be used for upcoming analyses dealing with high-dimensional gene expression data.

## Introduction

An important problem in toxicology in the context of gene expression microarray data is the simultaneous inference of a large number of concentration–response relationships. While gene expression of each gene is observed individually within the concentration–response experiments, the corresponding different experimental conditions, i.e. the concentrations, have to be the same for all genes. Therefore, the crucial question is how the different conditions should be allocated in order to reduce the costs of the expensive gene expression experiments while achieving a high informativeness of the resulting data.

Concerning the statistical analysis based on these experiments, the concentration–response relationships are typically modelled by nonlinear parametric curves to obtain certain parameters of interest for the individual genes, such as alert concentrations or the effect of a prespecified concentration on certain genes (see [1–3], among many others). Moreover, in each of these cases, the quality of the described inference substantially depends on the choice of the set of different conditions, at which observations are taken simultaneously for all genes. Thus, this paper is devoted to the construction of efficient sets of experimental conditions, also called optimal designs, that can be used for the simultaneous inference of a large number of nonlinear concentration–response curves.

Although determining optimal experimental designs for parametric curves has found considerable interest in the literature for the individual analysis of concentration–response relationships [4] and especially for the sigmoid Emax model [5–7], there is only a few literature on optimal design theory for multiple parametric curves. Dette and Schorning [8] constructed designs for the comparison of two different concentration–response curves, whereas Feller et al. [9] determine Determinant-optimal (*D*-optimal) designs for several concentration–response curves which share some parameters, but in both cases the resulting optimal designs are individually determined for each curve under consideration. Dror and Steinberg [10] developed robust designs based on a *K*-means algorithm for multiple experiments, but in the context of multivariate generalized linear models, which cannot directly be adapted to the situation considered in this paper. In the setting of high-dimensional microarray data, Dong et al. [11] introduced a procedure to obtain the maximally informative next experiment (MINE) for a high-dimensional linear model, in which the number of model parameters exceeds the sample size significantly. Recently, MINE was further investigated and extended to nonlinear models by Bouffier et al. [12] as well as McGee and Buzzard [13], respectively. Nevertheless, the MINE procedure is not applicable to the setting of this paper, as neither sequential approaches nor high-dimensional model parameters are included.

Instead, the design criteria developed in this paper aim for non-sequential designs that are efficient for all parametric concentration–response curves in terms of *D*-optimality. Therefore, the resulting designs for simultaneous inference contain information both about the allocation of the concentrations and about the relative amount of observations that should be taken at each concentration, respectively. In particular, we construct a *D*-optimality criterion for simultaneous inference by adapting the approach of Bayesian optimality criteria introduced by Chaloner [14] in combination with *D*-efficiencies (see [15], among many others). Although the resulting criterion is similar to the ones used in the case of one concentration–response curve, where the uncertainty of the true parameter is incorporated, its target is different, as it addresses the precise simultaneous inference of a large number of different curves. In a second approach, we use the large set of locally *D*-optimal designs obtained by considering the different curves individually and construct a cluster design based on a *K*-means cluster algorithm. In contrast to [10] the resulting algorithm is based on the approximate designs and directly applicable to the situation of concentration–response modelling.

Further, we demonstrate that the resulting optimal designs yield a substantially more accurate statistical analysis, using an application of our developed methods on a gene expression data set provided by Krug et al. [16]. In this study the expression level of 54,675 genes exposed to valproic acid (short: VPA) is measured at eight different concentrations (including placebo), and we show that using the developed optimal designs results in significantly more precise model fits of the concentration–response curves than using the eight original concentrations. Moreover, we compare the optimal designs with commonly used designs in practice, in particular with the equidistant and log-equidistant design. Thereby, we illustrate that the log-equidistant design is clearly insufficient for such analyses, while the equidistant design performs well.

The criteria developed in this paper depend on prior knowledge on the genes, in particular on the distribution of the corresponding nonlinear parameters within the concentration–response modelling. Possible application scenarios are experiments where prior knowledge is available either from previous experiments or from results on similar experiments given in the literature. In the latter setting, our approach is especially useful when the aim of the planned experiment is the reproduction of a former experiment in another laboratory.

Furthermore, a huge advantage of our approach is its flexibility. If a different substance with different characteristics is of interest, our method is directly adjustable to the new setting. Besides there is no restriction on the prior knowledge used for the developed criteria. For example, if only specific previously known gene groups are of interest this can be incorporated in the prior distribution in order to construct a design for simultaneous inference for these specific gene groups.

The remaining paper is structured as follows. First we introduce the situation under consideration and present two new methods for constructing optimal designs for simultaneous inference in the section “Methods”. Then we evaluate the performance of the developed methods on a real data example (VPA data set, see [16]). While the section “Designs for simultaneous inference of VPA-data” provides a detailed explanation of the construction of optimal designs based on this data set, in the section “Comparison of the designs” the performance of these new designs is compared to the original design and to commonly used designs in practice, in particular the equidistant and log-equidistant design. Here, we both analyse the theoretical efficiencies of the different designs under consideration and the results of an extensive simulation study. Finally, some conclusions and an outlook are given.

## Methods

### Classical optimal design theory

We assume that the same parametric model can be used to fit a curve describing the concentration–response relationship of each gene. More precisely, we assume that the data of each gene can be described by a nonlinear regression model

1 $$\begin{aligned} Y_{ij} = \eta (x_i,\theta ) + \varepsilon _{ij} \quad j=1,\ldots , r_i;\quad i=1, \ldots , n , \end{aligned}$$

where $\varepsilon _{ij}$ are independent centered normally distributed random variables, that is, $\varepsilon _{ij}\sim \mathcal{N}(0, \sigma ^2)$. This means that observations are taken at *n* concentrations $x_1, \ldots , x_n$ which vary in the design space $\mathcal{X}=[0, x_{\max }]\subset \mathbb{R}$ and $r_i$ observations $Y_{i1},\ldots , Y_{ir_i}$ are taken at each concentration $x_j$ ($j = 1,..., n$). Moreover, let $N=\sum _{i=1}^{n}r_i$ denote the total sample size. In general, a regression model $\eta (\cdot , \theta )$ with a *p*-dimensional parameter vector $\theta \in \Theta \subset \mathbb{R}^{p}$ is used to describe the dependence between the concentration (of a toxic compound) and the response.

Following Kiefer [17], we define an approximate design $\xi$ as probability measure with mass $w_i$ at the different support points $x_i\in \mathcal{X}$, which we denote by

$$\begin{aligned} \xi =\begin{pmatrix} x_1 &{}\ldots &{} x_n\\ w_1 &{} \ldots &{} w_n \end{pmatrix}. \end{aligned}$$

If an approximate design is given and *N* observations can be taken, a rounding procedure [18] is applied to obtain integers $r_i$ ($i=1, \ldots , n$) from the not necessarily integer values quantities $w_iN$, respectively. Then, under common assumptions of regularity and the assumption $\lim _{N\rightarrow \infty } \frac{r_i}{N}= w_i$ ($i=1, \ldots , n$) the maximum likelihood estimator $\hat{\theta }=(\hat{\theta }_1,\ldots , \hat{\theta }_p)^T$ satisfies

$$\begin{aligned} \sqrt{N}(\hat{\theta }-\theta ) \xrightarrow {\mathcal{D}} \mathcal{N}(0,\sigma ^2 M^{-1}(\xi , \theta )) \end{aligned}$$

as $N\rightarrow \infty$, where the symbol $\xrightarrow {\mathcal{D}}$ denotes weak convergence. The matrix $M(\xi , \theta )$ is called information matrix of the design $\xi$ and is defined by

$$\begin{aligned} M(\xi ,\theta )= \int _{\mathcal{X}}\frac{\partial }{\partial \theta } \eta (x,\theta )\left( \frac{\partial }{\partial \theta } \eta (x,\theta )\right) ^T d\xi (x), \end{aligned}$$

where $\frac{\partial }{\partial \theta } \eta (x,\theta )$ is the gradient of the regression function $\eta (x, \theta )$ with respect to the parameter $\theta \in \mathbb{R}^{p}$. Note that the gradient $\frac{\partial }{\partial \theta } \eta (x,\theta )$ depends on the unknown but true parameter vector, if the considered model $\eta (x, \theta )$ is nonlinear. The information matrix $M(\xi , \theta )$ is a measure of the information gained if the design $\xi$ is used. Consequently, designs that result in a large information matrix $M(\xi , \theta )$ in some sense, are appropriate. In practice, several criteria are measuring the quality of a design regarding the resulting information matrix and one of the most popular ones is the *D*-optimality criterion (see [17]). To be precise, a design $\xi ^*_{{\theta }}$ is called locally *D*-optimal, as it is proposed by Chernoff [19], for estimating the parameter $\theta$ when it maximizes the concave functional

2 $$\begin{aligned} \psi _D(\xi , \theta ) = \det (M(\xi , \theta ))^{1/p} \end{aligned}$$

among all designs $\xi$ on the design space $\mathcal{X}$, indicating the dependence of the *D*-optimal design on the parameter $\theta$. One key advantage of working with approximate designs and concave criteria is that convex optimization theory can be applied. As a consequence, a general equivalence theorem is available to verify whether a design is locally optimal among all designs. In particular, the locally *D*-optimality of a design $\xi ^*_{{\theta }}$ can be validated by checking whether the inequality

3 $$\begin{aligned} d(x,\xi ^*_{{\theta }},\theta )= \frac{\partial }{\partial \theta } \eta ^T(x, \theta )M^{-1}(\xi ^*_{\theta },\theta )\frac{\partial }{\partial \theta }\eta (x, \theta ) - p \le 0 \end{aligned}$$

is satisfied for all $x\in \mathcal{X}$ (see [20]).

In order to investigate the quality of a (non-*D*-optimal) design $\xi$, we consider its *D*-efficiency which is defined by

4 $$\begin{aligned} \text{Eff}_{D}\left( \xi ,\theta \right) =\frac{\psi _D(\xi ,\theta )}{\psi _D(\xi ^*_{\theta },\theta )} \, \end{aligned}$$

(see [21], among many others), and whose value is within the interval [0, 1] by definition. The better the design $\xi$ in terms of the *D*-optimality criterion $\psi _D$ is, the greater is its *D*-efficiency. Note that the *D*-efficiency also depends on the unknown parameter $\theta$, if the model in (1) is nonlinear.

### Optimal designs for simultaneous inference

In the situation of the paper, let $G\in \mathbb{N}$ be the number of different genes considered in the experiment. Then *G* corresponding different concentration–response curves (with different true parameter vectors) of the form (1) have to be fitted simultaneously using the same design $\xi$. Due to the dependence of the locally *D*-optimal designs on the unknown parameters, there is no locally *D*-optimal design which is appropriate for the simultaneous estimation of the *G* different curves. In particular, let $\theta ^{(1)} \ne \theta ^{(2)}$ be two parameter vectors of two different curves. Then the *D*-efficiency of the locally *D*-optimal design $\xi ^*_{\theta ^{(1)}}$ might be low if used for estimating the curve where $\theta ^{(2)}$ is the actual true parameter vector and vice versa.

Consequently, a design is required that provides high *D*-efficiencies for all *G* parameter vectors and we introduce an optimality criterion that addresses this need. More precisely, let $\pi$ be a discrete distribution on the set $\Theta _G$ which contains the different parameter vectors of the *G* different considered curves. Then a design $\xi ^*_{\Theta _G}$ is called *D*-optimal for the simultaneous inference (short version: simultaneous *D*-optimal) if

5 $$\begin{aligned} \Psi (\xi , \pi )= \sum _{\theta \in \Theta _G} \pi (\theta ) {\text{Eff}_{D}\left( \xi ,\theta \right) ,} \end{aligned}$$

is maximized by $\xi ^*_{\Theta _G}$ among all designs $\xi$ on the design space $\mathcal{X}$. Moreover, it can be checked if a given design $\xi$ is simultaneous *D*-optimal by checking whether the inequality

6 $$\begin{aligned} s(x, \xi , \pi )= \sum _{\theta \in \Theta _G} \pi (\theta ) \text{Eff}_{D}\left( \xi ,\theta \right) d(x, \xi , \theta ) \le 0 \end{aligned}$$

is satisfied for all $x\in \mathcal{X}$, where the function $d(x, \xi , \theta )$ is defined in (3). A Proof of this statement is given in the Additional file 1. Note that criteria that are of similar form as (5) were first introduced by Pronzato and Walter [22], Chaloner [14] as well as Chaloner and Larntz [23] and are also known as Bayesian or robust optimality criteria. These criteria are classically used if there is less knowledge about the unknown parameter value of one parametric curve which should be estimated. In this case a prior distribution, $\pi$, on the parameter space $\Theta$ is used to average the locally *D*-optimality criterion given in (2) with respect to different parameter values (see [24, 25], among others). Although the criteria are similar to the one defined in (5), we emphasize that the target of the latter mentioned is the finding of a design that results in good efficiencies for the *G* different parametric curves. In particular, the simultaneous *D*-optimality criterion prevents being affected by different sizes of the *G* different information matrices by standardizing via the *D*-efficiencies.

Also, the simultaneous *D*-optimality criterion becomes more complex, the more complex the set $\Theta _G$ and the corresponding distribution $\pi$ are. This might result in numerical problems when the corresponding simultaneous *D*-optimal design has to be calculated for a great number *G* of different parametric curves. One approach is the reduction of the support of the distribution $\pi$ to a smaller set $\tilde{\Theta }\subset \Theta _G$ that represents the complete set $\Theta _G$ appropriately.

Another approach for the construction of an appropriate design in the situation of simultaneous inference is motivated by a *K*-means cluster algorithm originally proposed by Hartigan and Wong [26]. More precisely, denote the support of a design $\xi$ by $\text{ supp }(\xi )$ and let $\text{ supp }(\xi _g^*)$ be the support of the locally *D*-optimal design for estimating the parameter $\theta ^g$, $\theta ^g \in \Theta _G$. Moreover, denote the intersection of all supports by ${C}_0 = \cap _{g=1}^{G} \text{ supp }(\xi _g^*)$ and the union of all supports by $C = \cup _{g=1}^{G} \text{ supp }(\xi _g^*)$. Fixing the number of different experimental conditions to $L\in \mathbb{N}^{\ge p}$, the *K*-means design with *L* different experimental conditions is determined in four consecutive steps:

1. Determine $\tilde{L}\le L$ different elements $c_1, \ldots , c_{\tilde{L}}$ in $C_0$ and set $K = L - \tilde{L}$.
2. Divide the set $C\setminus C_0$ into *K* disjoint sets $C_1, \ldots , C_K$ that satisfy $\cup _{k=1}^{K} C_k = C\setminus C_0$, using the *K*-means algorithm with Euclidean distance. Moreover, calculate the center of the set $C_k$ by
  $$\begin{aligned} \bar{c}_k = \frac{1}{\# C_k}\sum _{c_k\in C_k} c_k , \end{aligned}$$
  for $k=1, \ldots ,K$, respectively, where $\#A$ denotes the number of different elements in a discrete set *A*.
3. Repeat the second step *J* times. In the *j*-th step, sort the resulting cluster centers and denote them by $\bar{c}_{j\,(1)}< \cdots < \bar{c}_{j\,(K)}$. Calculate the mean of the *k*-th ordered center by
  $$\begin{aligned} \tilde{c}_{k}=\frac{1}{J} \sum _{i=1}^J \bar{c}_{i\,(k)} \end{aligned}$$
  for all $$k=1, \ldots , K$$.
4. The *K*-means design with *L* different experimental conditions is given by the probability measure with equal masses $\tfrac{1}{L}$ at the different experimental conditions $c_1, \ldots , c_{\tilde{L}}$ (see 1. step) and $\tilde{c}_1,\ldots , \tilde{c}_K$ (see 3. step). It is denoted by
  $$\begin{aligned} \xi _L=\begin{pmatrix} c_1 &{} \cdots &{} c_{\tilde{L}} &{} {\tilde{c}_1} &{} \cdots &{} {\tilde{c}_K}\\ \frac{1}{L} &{} \cdots &{} \frac{1}{L} &{} \frac{1}{L} &{} \cdots &{} \frac{1}{L} \end{pmatrix}. \end{aligned}$$

Equal weights are used for the *K*-means design with *L* experimental conditions $\xi _{L}$ for simplicity, other weights that incorporate the distribution of the different parameter values of the *G* different parametric curves can also be used. The third step of the algorithm is included to obtain robustness with respect to the resulting clusters. Moreover, the algorithm can be further improved by using methods provided by Apon et al. [27] among many others.

Note that Dror and Steinberg [10] proposed a similar heuristic approach for constructing robust exact optimal designs based on a *K*-means algorithm for multivariate generalized linear models. While [10] use a rough grid over the parameter space and exact designs, all considered model parameters are included in our approach. Moreover, our approach is based on the corresponding locally *D*-optimal approximate designs. While the number of clusters *K* is variable in the algorithm of [10], we fix the number of clusters in advance to a fixed *K*.

Although the *K*-means design is based on locally *D*-optimal designs, and therefore on convex design criteria, it is not possible to derive sufficient and necessary conditions of the form (6) to check the optimality of the *K*-means design.

### The sigmoid Emax model

In the context of gene expression data, the concentration–response relationship often shows a sigmoidal course. Therefore, the regression model in (1) is frequently used with the sigmoid Emax function as regression function $\eta (\cdot , \theta )$ (see [28]). For a concentration $x \in \mathcal{X} = [0, x_{\max }]$ and a parameter vector $\theta =(\text{E}_0,\text{E}_{\text{max}}, \text{EC}_{50}, h)\in \mathbb{R}^4$, the sigmoid Emax function is defined by

7 $$\begin{aligned} \eta (x, \theta ) = \text{E}_0+ \frac{x^h\cdot \text{E}_{\text{max}}}{x^h+{\text{EC}^h_{50}}}, \end{aligned}$$

where the parameter $\text{E}_0$ describes the effect at the placebo concentration, $x=0$, and the parameter $\text{E}_{\text{max}}$ specifies the maximal effect associated with the considered compound. Moreover, the parameter $\text{EC}_{50}$ denotes the mean effective concentration, which describes the concentration at which 50% of the maximal effect associated with the compound is attained and the hill parameter *h* quantifies the slope of the regression function [28].

The sigmoid Emax function in (7) is nonlinear in the parameters $\text{EC}_{50}$ and *h* such that its information matrix $M(\xi , \theta )$ and the locally *D*-optimal design $\xi ^*$ depend on these parameters. Due to the complexity of the sigmoid Emax function, the locally *D*-optimal designs maximizing the criterion in (2) cannot be determined analytically. However, results are available about the structure of the locally *D*-optimal design: In particular, Wang and Yang [7] proved that the locally *D*-optimal design consists of four different support points, while Li and Majumdar [6] derived that two of these support points equal the boundary points of the design space $\mathcal{X}=[0, x_{\max }]$. Using Lemma 5.1.3. stated in [29] the locally *D*-optimal design $\xi ^*$ for the sigmoid Emax model is of the form

$$\begin{aligned} \xi ^*=\begin{pmatrix} 0&{}x_2&{} x_3&{} x_{\max }\\ 0.25&{}0.25&{}0.25&{}0.25 \end{pmatrix}, \end{aligned}$$

where $x_2, x_3 \in \mathcal{X}$ have to be calculated numerically in dependence on $\theta$.

## Designs for simultaneous inference of VPA-data

In the following sections, the construction of designs for simultaneous inference is illustrated by an application on a gene expression data set, called VPA-data [16]. In the section “Data”, the VPA-data set is described, whereas the section “Data preprocessing and analysis” provides a description of the initial data preprocessing and analysis steps. In the section “Construction of K-means and D-optimal design for simultaneous inference”, we construct both the *K*-Means design and the simultaneous *D*-optimal design aiming for a precise inference of the concentration–response relationships of the VPA-data. All analyses were performed using the statistical software R, version 4.2.2 [30].

### Data

In a study proposed by Krug et al. [16] human embryonic stem cells were exposed to valproic acid (short: VPA). Originally the neurotoxicity was evaluated by conducting experiments with Affymetrix Human Genome U133 Plus 2.0 gene chips. The gene expression values for 54,675 probe sets were evaluated in a crude form. For the sake of clarity, the probe sets are simply considered as genes in the following. The design space $\mathcal{X}$ of potential concentrations is given by $\mathcal{X} = [0, 1000]$ and the responses were measured at the concentrations 25, 150, 350, 450, 550, 800, and 1000 $\mu$M conducted three times with different experiments. Additionally, the placebo concentration 0 was executed six times. Thus, 27 measurements at 8 different concentrations are available for each gene. The corresponding approximate design denoted as original design $\xi _{\text{orig}}$ is given by

$$\begin{aligned} \xi _{\text{orig}}=\begin{pmatrix} 0&{}25 &{} 150 &{} 350 &{} 450 &{} 550 &{} 800 &{} 1000\\ \frac{2}{9}&{}\frac{1}{9}&{}\frac{1}{9}&{}\frac{1}{9}&{}\frac{1}{9}&{}\frac{1}{9}&{}\frac{1}{9}&{}\frac{1}{9}\\ \end{pmatrix}. \end{aligned}$$

### Data preprocessing and analysis

First, the data of the 54,675 genes are examined with respect to their biological activity. More precisely, we use the multiple contrast test of the multiple contrast procedure method (MCP-Mod) introduced by Bretz et al. [31] to detect genes whose concentration–response relationship follows a sigmoid Emax model defined by (7) to ensure a convenient model fit. Although we are reducing the data set we are not interested in a dimensionality reduction of the data set or identifying specific gene sets. Such approaches have been proposed by Azadifar et al. [32] and Rostami et al. [33], but are not considered in this paper. The MCP-Mod procedure requires specifications for the nonlinear parameter values of the used model, which are the $\text{EC}_{50}$ and *h* parameter in case of the sigmoid Emax model. Following Duda et al. [34], we fix these parameters to $\text{EC}_{50}=450$ and $h=5.118$. Note that no adjustment for multiple testing was conducted. In contrast to multiple testing procedures, which aim at controlling the type I error, our goal is to develop and analyse methods on a huge number of genes. In doing so we want to identify all genes with a sensible sigmoidal model course. Thus we set the significance level to $\alpha = 0.01$. According to the MCP-Mod procedure 33,884 genes are not significant, which implies these cannot be modelled properly by the sigmoid Emax model and biological activity cannot be assumed for these genes. For 20,791 genes, the sigmoid Emax model is significant, which indicates that biological activity and a convenient sigmoid Emax model fit can be assumed.

We now concentrate on the analysis of the remaining 20,791 genes and the corresponding concentration–response relationships. In particular, we fit a regression model of the form (1) with the sigmoid Emax function (7) to the data of each gene using maximum-likelihood estimation. The estimation is provided by the function fitMod contained in the R-package DoseFinding [35] with corresponding predefined parameter restrictions for the nonlinear parameters $\text{EC}_{50}\in [0, 1500]$ and $h\in [0.05, 10]$. In the case of a fitted $\text{EC}_{50}> 1000$, the concentration at which $50\%$ of the maximal effect is attained lies outside the design space $\mathcal{X}=[0, 1000]$. From the point of view of experimental design, the constellation of an $\text{EC}_{50}$-value outside the design space is not reasonable and will result in an insufficient model fit independent from the design. In particular, the model fits will nevertheless aim for an estimate of $\text{EC}_{50}$ inside the design space. Thus, we restrict ourselves to the analysis of the concentration–response curves whose estimated parameter $\hat{\theta }$ is contained in the parameter space $\Theta = \mathbb{R} \times \mathbb{R} \times [0, 1000] \times [0.05, 10]\subset \mathbb{R}^4.$ Furthermore, we removed data of other 85 genes, as their parameter combinations lead to numerical instabilities in the further analysis. Summarizing, the data set is reduced to $G=15{,}233$ genes and we store the corresponding parameter estimates of the data set in a reduced parameter space $\Theta _G$, defined by

8 $$\begin{aligned} \Theta _G =\{\theta ^g= (\hat{\text{E}}_0, \hat{\text{E}}_{\max }, \hat{\text{EC}}_{50}, \hat{h})^T\,\,|\exists g\in \{1, \ldots , 15233\}: \theta ^g \in \Theta \} . \end{aligned}$$

**Fig. 1:**
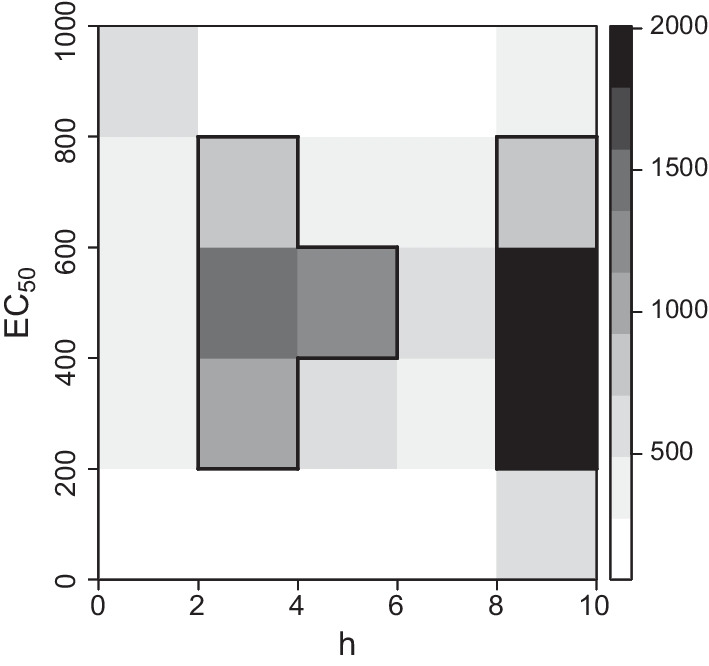
Heatmap of steepness *h* and $\text{EC}_{50}$ of $\tilde{\Theta }_G$, encircled areas correspond to frequencies higher 5%

We now focus on the distribution of the nonlinear parts of the parameter estimations, that is $(\text{EC}_{50},h)^T$ and analyse the distribution of the values contained in the two-dimensional set

9 $$\begin{aligned} \tilde{\Theta }_G = \{(\hat{\text{EC}}_{50}, \hat{h})^T\,\,|\,\, \exists g\in \{1, \ldots , 15233\}: \theta ^g \in \Theta \} . \end{aligned}$$

on $\tilde{\Theta }= [0, 1000] \times [0.05, 10]$. Figure 1 illustrates the distribution of the steepness *h* and the $\text{EC}_{50}$ of every fitted sigmoid Emax model on $\tilde{\Theta }$ displaying a $\left( 5\times 5\right)$-grid classification on $\tilde{\Theta }$ of the parameter estimates contained in $\tilde{\Theta }_G$.

Due to the contrast levels an accumulation for genes with high steepness parameters independent of the ${\text{EC}}_{50}$-value is clearly visible through the darker areas. In particular, for 31.7% of all genes the estimated steepness parameter is given by $\hat{h} = 10$, which is the upper bound for *h* within the parameter space $\Theta$. For $56.2\%$ of the considered genes it holds $\hat{h} \in (2, 10)$. Concentrating on the $\text{EC}_{50}$-values (independent from *h*), 84.5 % of the estimates take values in the interval (200, 800). Both small values i.e. $\hat{EC}_{50} \le 200$, and high values, i.e. $\hat{EC}_{50}>800$, are rarely present (7.7% and 7.8%).

Note that each of the seven areas in Fig. 1, which are encircled in black, indicates a frequency of more than 5% and the union of these seven areas constitutes 61% of the parameter estimates contained in $\Theta _G$. Therefore, we concentrate on the analysis of these areas, which are listed with corresponding representative estimates in Table 1. Note that the representative parameter estimates were drawn randomly from each of the seven areas, respectively, and that we denote the set of these vectors by $\Theta _7=\{\theta ^1, \ldots , \theta ^7\}$.

**Table 1:** Representative parameter vectors $\theta ^i$ of parameter set $\Theta _7$ and corresponding weight distribution $\pi _7$ for the representated areas with frequencies higher than $5\%$

Representated Area	$\Theta _7$	$\text{EC}_{50}$	*h*	$\pi _7(\theta ^g)$
$(200, 400] \times (2, 4]$	$\theta ^1$	298.81	2.53	0.1192
$(400, 600] \times (2, 4]$	$\theta ^2$	575.00	3.77	0.1633
$(600, 800] \times (2, 4]$	$\theta ^3$	758.84	2.72	0.0897
$(400, 600] \times (4, 6]$	$\theta ^4$	469.36	4.00	0.1225
$(200, 400] \times (8, 10]$	$\theta ^5$	310.60	10.00	0.2009
$(400, 600] \times (8, 10]$	$\theta ^6$	501.97	10.00	0.2172
$(600, 800] \times (8, 10]$	$\theta ^7$	747.88	10.00	0.0872

**Fig. 2:**
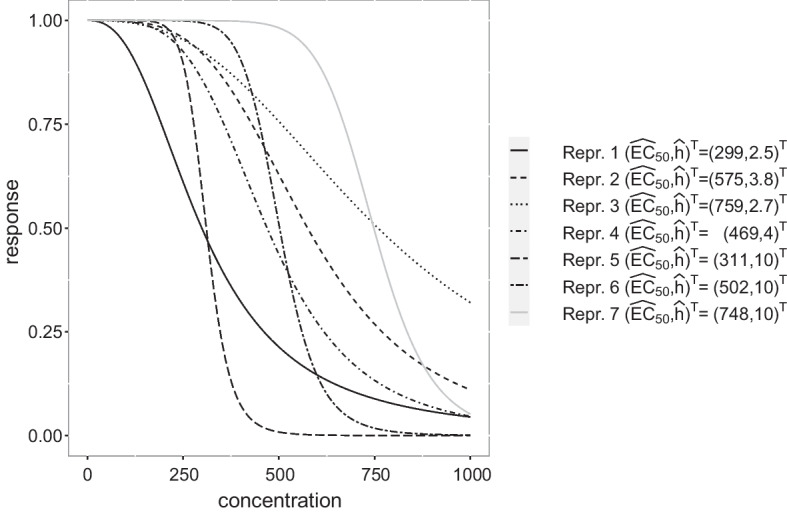
Sigmoid Emax model fits to the representative parameter vectors given in Table 1 where the linear parameters are fixed to $\text{E}_0=1$ and $\text{E}_{\max } = -1$, respectively

In Fig. 2 the resulting estimated sigmoid Emax curves of the seven representative parameter estimates are shown, where the $E_0$ and $E_{\max }$ values are set to 1 and $-1$, respectively, for the sake of comparability. We observe that the representative parameter estimates result in both curves that are saturated within the design space $\mathcal{X} = [0, 1000]$ (i.e. representatives 4 and 6) and in curves that are still significantly decreasing at the upper bound of the design space $\mathcal{X}$ (see representative 1, 2, 3). Thus, the determination of a design that is suitable for the joint estimation of these different curves is demanding and we concentrate on that task in the following section.

### Construction of *K*-means and *D*-optimal design for simultaneous inference

For the construction of the *K*-means design and the simultaneous *D*-optimal design, the locally *D*-optimal designs based on the $G=15{,}233$ different parameter estimations in $\Theta _G$ are necessary. Moreover, the locally *D*-optimal designs for these parameters are needed for the calculation of the *D*-efficiencies defined by (4). Consequently, we first determined the locally *D*-optimal designs using the results of its principle structure stated in the section “The sigmoid Emax model”. In particular, in the present situation the locally *D*-optimal design $\xi _{\text{Dopt}}^g$ for a particular parameter $\theta ^g=(\hat{E}_0, \hat{E}_{\max }, \hat{E}_{50}, \hat{h})^T\in \Theta _G$ is of the form

$$\begin{aligned} \xi _{\text{Dopt}}^g=\begin{pmatrix} 0 &{} x_2 &{} x_3 &{} 1000\\ 0.25 &{} 0.25 &{} 0.25 &{} 0.25\\ \end{pmatrix}. \end{aligned}$$

The additional support points $x_2, x_3$ were calculated numerically in dependence of $\theta ^g$ using the particle swam optimization-algorithm (PSO), which is a heuristic optimization algorithm (see [36] for details). To check the *D*-optimality of the designs obtained by PSO, the inequality of the corresponding Equivalence Theorem given in (3) was checked, respectively. Note again that it holds $\xi _{\text{Dopt}}^g\ne \xi _{\text{Dopt}}^{g'}$ for $\theta ^g \ne \theta ^{g'}$, in general and a design that is *D*-optimal for $\theta ^g$ might not be appropriate for the parameter $\theta ^{g'}$ (see the section “Optimal designs for simultaneous inference” and “The sigmoid Emax model” for details).

For the construction of an equally weighted *K*-means design, we follow the procedure described in the section “Optimal designs for simultaneous inference”. Imitating the property of the original design given in Table 2, which has 8 support points with the placebo concentration weighted twice, the total number of different support points for the *K*-means design is fixed to $L=9$. Due to the structure of the 15,233 different locally *D*-optimal designs the intersection of all supports is given by $C_0 = \{0, 1000\}$, such that $\tilde{L} = 2$. Thus, the clustering is done for $K=9-2=7$ clusters on the set $C\setminus C_0$ which contains the support points of the different locally *D*-optimal designs without the concentrations 0 and 1000. The resulting equally weighted *K*-means design with 9 support points (short: *K*-means design) can be found in Table 2 where the support points are rounded to integers for the sake of readability.

**Table 2:** Considered designs with information of used concentrations and corresponding weights

Design	Notation	Concentrations and corresponding weights								
Original	$\xi _{\text{orig}}$	0	25	150	350	450	550	800		1000
		$\frac{2}{9}$	$\frac{1}{9}$	$\frac{1}{9}$	$\frac{1}{9}$	$\frac{1}{9}$	$\frac{1}{9}$	$\frac{1}{9}$		$\frac{1}{9}$
Equidistant	$\xi _{\text{equi}}$	0	125	250	375	500	625	750	875	1000
		$\frac{1}{9}$	$\frac{1}{9}$	$\frac{1}{9}$	$\frac{1}{9}$	$\frac{1}{9}$	$\frac{1}{9}$	$\frac{1}{9}$	$\frac{1}{9}$	$\frac{1}{9}$
Log-equidistant	$\xi _{\text{log}}$	0	1	3	7	19	52	139	373	1000
		$\frac{1}{9}$	$\frac{1}{9}$	$\frac{1}{9}$	$\frac{1}{9}$	$\frac{1}{9}$	$\frac{1}{9}$	$\frac{1}{9}$	$\frac{1}{9}$	$\frac{1}{9}$
*K*-means	$\xi _{\text{kmeans}}$	0	89	209	326	428	536	652	798	1000
		$\frac{1}{9}$	$\frac{1}{9}$	$\frac{1}{9}$	$\frac{1}{9}$	$\frac{1}{9}$	$\frac{1}{9}$	$\frac{1}{9}$	$\frac{1}{9}$	$\frac{1}{9}$
Simultaneous *D*-optimal	$\xi _{\Theta _7}$	0	145	280	345	457	575	656	781	1000
		0.17	0.05	0.12	0.12	0.11	0.14	0.03	0.06	0.20

For the determination of the simultaneous *D*-optimal design in (5), a discrete distribution $\pi$ on $\Theta _G$ is necessary. The choice of the uniform distribution on the parameter set $\Theta _G$ given by (8) is not appropriate in the case under consideration, as the number of different parameter estimates contained in this set is huge ($G=15{,}233$) and the corresponding *D*-optimality criterion for simultaneous inference becomes numerically instable. Therefore, we recall the seven areas and the corresponding representative parameter estimates presented in Table 1 instead. We set the support of the considered distribution $\pi$ to the set $\Theta _7=\{\theta ^1, \ldots , \theta ^7\}$, which are the representative parameter estimates of the seven significant parameter areas (see Table 1). For $\theta \in \Theta _7$, the probability $\pi (\theta )=\pi _7(\theta )$ is set to the readjusted relative frequency of the corresponding area, which is listed in the last column of Table 1. For $\theta \in \Theta _G \setminus \Theta _7$, it then follows $\pi _7(\theta ) = 0$.

Using PSO based on the distribution $\pi _7$, we obtain the simultaneous *D*-optimal design given in Table 2 where the support points are again rounded to integers for the sake of readability. Note that the optimality of the design $\xi _{\Theta _7}$ can be checked by plotting the function $s(x, \xi _{\Theta _7}, \pi _7)$ given in (6) (see Additional file 1: Figure S1).

In the section “Comparison of the designs”, the *K*-means design, the simultaneous *D*-optimal design and the original design are compared concerning different measures of performance. Furthermore, we include an equidistant and a log-equidistant design with nine support points on the design space $\mathcal{X} = [0, 1000]$ in the analysis, since such designs are commonly used in the context of gene expression data (see [37, 38], among many others). While Pinheiro and Bornkamp [39] argued that the log-equidistant design is superior to the equidistant design if used for the analysis of one concentration–response curve, we investigate whether this also holds true in the context of the simultaneous analysis of gene expression data.

All designs under consideration are shown in Table 2, an illustration of them is contained in the Additional file 1: Figure S2.

## Comparison of the designs

In the following sections, the performances of the different designs depicted in Table 2 are investigated when they are used for the estimation of the 15,233 concentration–response curves. In the section “Comparison with respect to the D-efficiencies”, the designs are compared with respect to their *D*-efficiencies, whereas in the section “Comparison using a simulation study” the designs are used to simulate new concentration–response data for each of the 15,233 genes. Based on this data, new concentration–response curves are estimated and compared to the curves obtained by the original VPA-data.

### Comparison with respect to the *D*-efficiencies

As stated in the section “Optimal designs for simultaneous inference”, the performance of a given design $\xi$ can be measured using the *D*-efficiency $\text{Eff}_{D}(\xi , \theta )$ defined in (4), where $\theta \in \Theta$ is the assumed true parameter vector. The greater the *D*-efficiency is, the better the corresponding design performs.

**Fig. 3:**
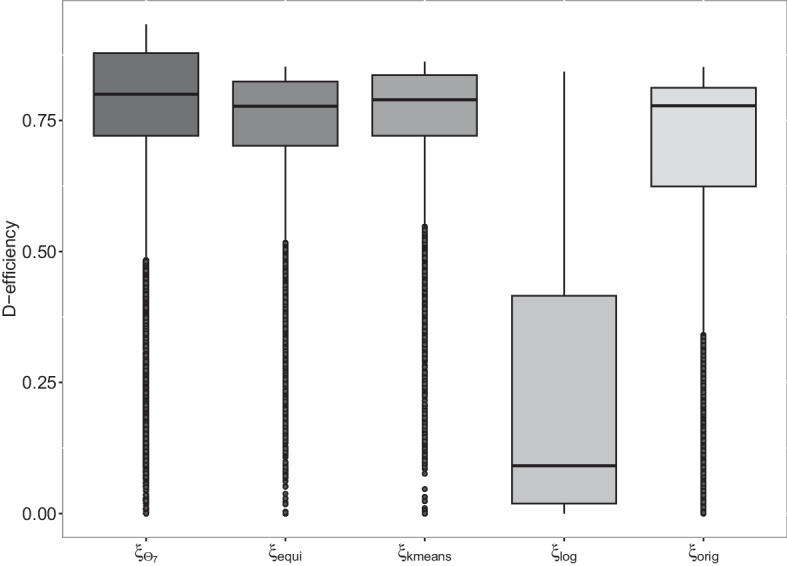
*D*-efficiencies of the different designs under consideration assuming the different parameter vectors $\theta \in \Theta _G$

We analyse the *D*-efficiencies of the designs depicted in Table 2 for all parameter vectors contained in $\Theta _G$, i.e. the parameter set containing the $G=15{,}233$ significant parameter vectors for the corresponding genes (see Eq. (8) and the section “Data preprocessing and analysis” for details). In Fig. 3, the resulting *D*-efficiencies are presented as box plots for each considered design, respectively. Whereas Table 3 depicts the corresponding benchmark *D*-efficiencies. i.e. their minima, maxima, quantiles, medians and means. Based on the *D*-efficiencies the log-equidistant design performs worst, whereas the simultaneous *D*-optimal design performs best.

**Table 3:** Descriptive parameters of the *D*-efficiencies regarding each design, minimum, 0.25%- and 0.75%-quantiles, median, mean and maximum

Design	Min	0.25%	Median	Mean	0.75%	Max
Simultaneous *D*-optimal	0.000	0.721	0.801	0.745	0.879	0.933
Equidistant	0.000	0.701	0.777	0.716	0.824	0.852
*K*-means	0.000	0.721	0.789	0.732	0.836	0.861
Log-equidistant	0.000	0.019	0.091	0.228	0.415	0.843
Original	0.000	0.624	0.778	0.682	0.812	0.852

More precise, the log-equidistant design $\xi _{\text{log}}$ has a *D*-efficiency smaller than 0.5 for more than $75\%$ of the parameter vectors contained in the parameter set $\Theta _G$ and a median *D*-efficiency of 0.09, which indicates a bad performance with respect to the *D*-optimality criterion for most of the considered parameter vectors.

In contrast 75% of the *D*-efficiencies of the simultaneous *D*-optimal design are greater than 0.72 and its median efficiency is equal to 0.80. In particular, the maximal *D*-efficiencies of the equidistant, original and *K*-means design are smaller than the 75%-quantile of the simultaneous *D*-optimal design, respectively. Thus, for 25% of all genes the simultaneous *D*-optimal design provides higher *D*-efficiencies than the maximal *D*-efficiency of the other designs, respectively. Nevertheless, the *K*-means design also results in high *D*-efficiencies, $75\%$ of the *D*-efficiencies are greater than 0.72 and its median is given by 0.79. The *D*-efficiencies of the equidistant design are similar to the *D*-efficiencies of the *K*-means design, which can be explained by the similar structure of these designs. Note that both designs are equally weighted and their support points are similarly distributed over the design space $\mathcal{X}=[0, 1000]$ (cf. Table 2 and Figure S2, see Additional file 1). Considering the original design, 75% of its *D*-efficiencies are greater than 0.62.

Note that there are several outliers for all designs under consideration. We investigated that parameter vectors that lead to very small *D*-efficiencies of the original design result in significantly small *D*-efficiencies of the other designs and almost vice versa. In particular, parameter combinations with $\text{EC}_{50} \le 200$ and large steepness $h> 8$ lead to small *D*-efficiencies independent from the design.

Although the simultaneous *D*-optimal design performs best, when the *D*-efficiencies are compared without restrictions, a worse performance could be possible for parameter vectors $\theta$, that are not part of the seven areas included in the distribution $\pi _7$. Therefore, we also investigate the *D*-efficiencies of the different designs grouped by the nonlinear parameters of the sigmoid Emax model, i.e. $\text{EC}_{50}$ and *h*. Analogously to the overall analysis the simultaneous *D*-optimal design shows the highest *D*-efficiencies for most of the groups: Only for a few parameter constellations, that were not considered in the construction of the design, lower *D*-efficiencies occurred. Regarding the remaining considered designs, the structure of their *D*-efficiencies was similar to the one in the overall analysis, respectively. A detailed description and additional figures of the grouped analysis can be found in the Additional file 1.

Summarizing the simultaneous *D*-optimal design performs best with respect to the *D*-efficiencies, whereas the log-equidistant design performs worst. The *K*-means and the equidistant design perform well, resulting in similar *D*-efficiencies for the considered cases. The original design results in *D*-efficiencies which are in principle allocated between the ones of the log-equidistant and the equidistant design.

### Comparison using a simulation study

In this section, we report the results of a simulation to investigate the performance of the different designs in Table 2 in scenarios that imitate concentration–response experiments for the 15,233 significant genes contained in the VPA-data set. In the section “Simulation study setup”, we introduce the design of the simulation study, including its assumptions and scenarios. Further, we describe the normalized root mean square error (NRMSE) which is used to evaluate the performance of the different designs within the study. In the section “Simulation results”, we summarize the results of the simulation study with respect to the NRMSE.

#### Simulation study setup

Imitating the original data set of Krug et al. [16], we investigate the performance of the five different designs if used for the simultaneous estimation of the concentration–response relationships corresponding to the 15,233 significant genes of the data set. We consider simulation parameters denoted in Table 4.

**Table 4:** Simulation parameters

Parameter	Variation/Value
Sample size	$N \in \left\{ 18,27,36,45,63,90\right\}$
Design	$\xi _{\text{orig}}$, $\xi _{\text{equi}}$, $\xi _{\text{logequi}}$, $\xi _{\text{kmeans}}$, $\xi _{\Theta _7}$
Gene	$g\in \left\{ 1,\ldots ,15233\right\}$
Model parameter	$\theta ^g\in \Theta _G =\left\{ \theta ^1,\ldots ,\theta ^{15{,}233}\right\}$
Simulation step	$j \in \left\{ 1,\ldots ,500\right\}$
$\sigma$	$0.2 \cdot | E^g_{\max }|$

More precisely, we consider six different sample sizes $N=18,27,36,45,63,90$ for each design given in Table 2. For the equidistant, log-equidistant and *K*-means design varying the sample size *N* leads to equal repetitions at every concentration, as these designs are equally weighted, whereas for the original design, there are twice as much repetitions at placebo ($x=0$) as at the remaining non-placebo concentrations. In case of unequal repetitions as attained for the simultaneous *D*-optimal design the procedure of efficient rounding according to Pukelsheim and Rieder [18] is used to obtain integer numbers of repetitions.

Further, we assume that the concentration–response relationship of each significant gene *g* is described by the nonlinear regression model (1), where the regression function is given by the sigmoid Emax model in (7) with the corresponding true parameter given by the estimate $\theta ^g \in \Theta _G$ given by (8). The errors in model (1) are assumed to be normally distributed with standard deviation $\sigma =0.2 \cdot | E^g_{\max }|$, where $E^g_{\max }$ is the maximal effect of gene *g*, respectively. This results in $\mathcal{S} = 5 \cdot 6 = 30$ different scenarios in total, and for each scenario, we obtain data from 15,233 concentration–response relationships.

We used $N_{\text{sim}}= 500$ simulation runs for each scenario and in each simulation step, the sigmoid Emax model is fitted to the data of each gene separately.

We use the Root Mean Squared Error (RMSE) to evaluate the performance of the different designs. For a given scenario *S* (out of the $\mathcal{S}=30$ scenarios), let $\eta (\cdot , \hat{\theta }^{g}_{jS})$ denote the estimated sigmoid Emax model with corresponding estimated model parameter $\hat{\theta }^{g}_{jS}$ for the data generated for gene *g* in simulation *j* ($g=1, \ldots , 15233$, $j=1,\ldots , 500$). Moreover, let $\eta (\cdot ,{\theta }^{g})$ denote the data generating sigmoid Emax model of the *g*-th significant gene. Following Cheema [40], the RMSE (of the *S*-th scenario for the *g*-th gene) is then given by

$$\begin{aligned} \text{RMSE}(g, S)=\frac{1}{N_{\text{sim}}} \sum _{j=1}^{N_{\text{sim}}} \sqrt{\frac{1}{1001}\sum _{i=0}^{1000}\left( \eta (x_i, \hat{\theta }^{g}_{jS})-\eta (x_i,{\theta }^{g})\right) ^2}, \end{aligned}$$

where $x_0, \ldots , x_{1000}$ are given by the sequence $0, 1, \ldots , 1000 \in \mathcal{X}$. There is high variability in the ranges of the expression values across genes, which is not accounted for by the RMSE. For example, if we consider two genes with the same RMSE value of 2, but with different response ranges, e.g. 4 and 10, the RMSE value for the gene with larger range shows a higher model precision compared to the gene with a smaller range, although this is not reflected directly by the RMSE. In order to obtain comparability between the curves associated to the different genes, it is useful to standardize the RMSE. This is achieved by dividing the RMSE by $\text{E}^g_{\max }$, which is the maximal range of the curve corresponding to gene *g*. Thus, it holds:

10 $$\begin{aligned} \text{NRMSE}(g, S) = \tfrac{\text{RMSE}(g, S)}{E_{\max }^{g}} . \end{aligned}$$

Note that the smaller the NRMSE is, the closer the fitted model is to the true concentration–response relationship.

#### Simulation results

In the section “Results of the different designs with fixed sample size”, we present the results for the different designs contained in Table 2, where the sample size is fixed to the sample size $N=27$, which coincides with the sample size of the original data set (see the section “Data” for details). In the section “Variation of sample size”, we analyse the influence of the sample size on the performance of the different designs.

#### Results of the different designs with fixed sample size

**Fig. 4:**
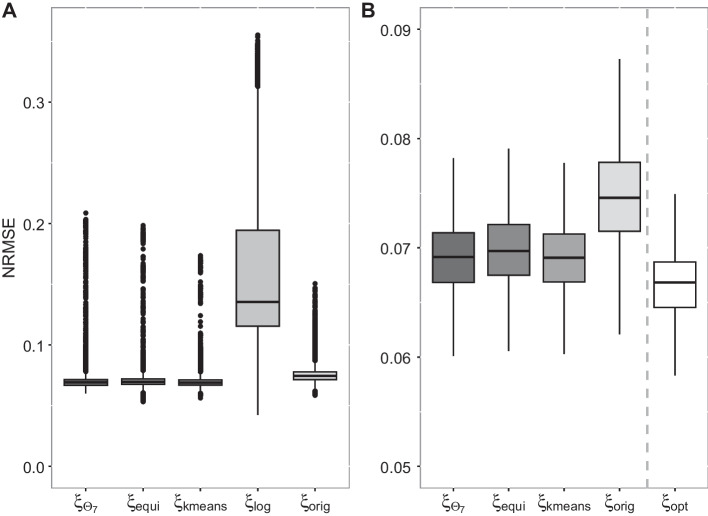
**A** NRMSEs for each gene with respect to the different designs. **B** NRMSEs for each gene with respect to the different designs, where the values of the log-equidistant designs and the outliers are removed

In the left part of Fig. 4, we display the NRMSE defined by (10) for the 15,233 curves with box plots grouped by the different designs under consideration. Also Table 5 depicts corresponding benchmark values of the NRMSEs like minima, maxima, quantiles, medians and means.

**Table 5:** Descriptive parameters of the *D*-efficiencies regarding each design, minimum, 0.25%- and 0.75%-quantiles, median, mean and maximum

Design	Min	0.25%	Median	Mean	0.75%	Max
Simultaneous *D*-optimal	0.060	0.067	0.069	0.071	0.071	0.209
Equidistant	0.053	0.068	0.070	0.070	0.072	0.198
*K*-means	0.056	0.067	0.069	0.070	0.071	0.174
Log-equidistant	0.042	0.116	0.135	0.161	0.194	0.355
Original	0.059	0.072	0.075	0.076	0.078	0.151
Locally *D*-optimal	0.053	0.065	0.067	0.067	0.069	0.147

It is clearly visible that the NRMSEs corresponding to the log-equidistant designs are greater than the NRMSEs of the other designs. In particular, almost 50% of the considered genes have an NRMSE, that is almost twice as large as for the other designs. Moreover, the NRMSEs obtained with the log-equidistant design, are extremely varying compared to the others. Thus, it follows that using the log-equidistant design is not reasonable for the data at hand and we restrict ourselves to the analysis of the NRMSEs obtained for the original, the equidistant, the *K*-means, and the simultaneous *D*-optimal design, respectively. For that purpose, we also remove the outliers, as no structure of these could be detected in dependence of the design choice. Further analyses resulted in observations similar to the ones for the *D*-efficiencies: In particular, outliers occur mostly for extreme parameter combinations such as $\text{EC}_{50}\le 2$ and $h> 8$.

The right part of Fig. 4 shows the NRMSEs without outliers grouped by all designs apart from the log-equidistant design. Additionally, the NRMSE of the locally *D*-optimal design of each gene *g* is provided, respectively. Note that the locally *D*-optimal designs, in general called $\xi _{\text{opt}}$, lead to the smallest NRMSEs and constitute the best choice for evaluating each gene separately. Nevertheless, these designs are not applicable in the experiment under consideration, which is done simultaneously for all genes. Consequently, the box plot of the NRMSEs based on the locally *D*-optimal designs has to be interpreted as benchmark.

Apart from the locally optimal designs, the best results with respect to the NRMSE are achieved by the simultaneous *D*-optimal design and the *K*-means, followed by the NRMSEs of the equidistant design. For instance, the median is 0.069 for both $\xi _{\Theta _7}$ and $\xi _{\text{kmeans}}$ and 0.070 for the equidistant design. These designs result in NRMSEs that are close to the ones of the locally *D*-optimal designs, which indicates a good performance with respect to the NRMSE. Finally, the original design results in larger NRMSEs than the other designs. In particular, the lower quartile of NRMSEs is given by 0.072, which is even greater than the upper quartiles of the equidistant, *K*-means, and simultaneous *D*-optimal design.

Similarly to the theoretical analysis of the *D*-efficiencies in the section “Comparison with respect to the D-efficiencies”, we investigated the NRMSEs grouped by the parameters $\text{EC}_{50}$ and *h*. Summarizing, the comparison of the RMSEs of the designs (stratified to different parameter constellations) leads to similar results as within the total analysis. A detailed description and corresponding figures can be found in the Additional file 1.

Summarizing, the designs constructed in the section “Construction of K-means and D-optimal design for simultaneous inference” outperform the original design with respect to the NRMSEs. Moreover, the equidistant design, which has support points similar to the ones of the *K*-means design, results in appropriate NRMSEs, whereas for the log-equidistant design NRMSEs are substantially higher in comparison to all other designs. Both the simultaneous *D*-optimal design and the *K*-means result in the best NRMSEs and therefore in the best simultaneous inference of the 15,233 concentration–response relationships.

#### Variation of sample size

**Fig. 5:**
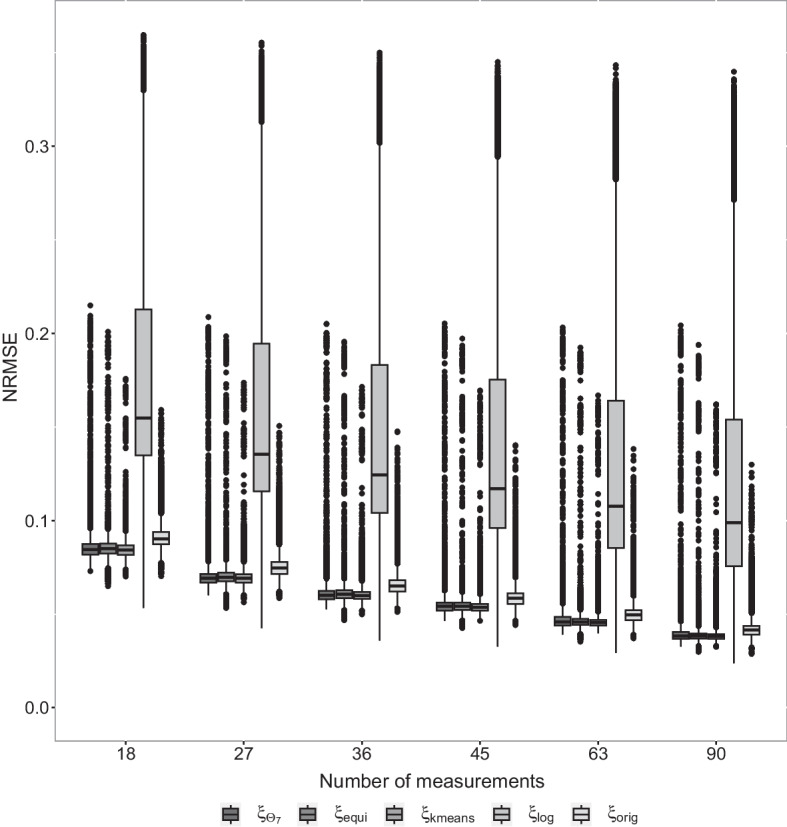
NRMSE values for each gene regarding different designs with varying number of measurements

We consider the NRMSEs of the different designs when the sample size *N* is varied, that is $N= 18, 27, 36, 45, 63, 90$. In Fig. 5, we display the NRMSEs grouped by design and the total sample sizes.

For all considered designs the NRMSEs are decreasing with increasing sample size, which implies an increase in the precision of the corresponding model fits. In particular, the median NRMSEs of all designs are located within 0.084 and 0.092 for $N=18$ measurements and decrease to values between 0.038 and 0.042 for $N=90$. Thus, the NRMSEs are almost reduced to half of the values by increasing the sample size, if the sample size is multiplied by 4. This effect is well explained by the convergence rate of the maximum likelihood estimator, which is $\sqrt{N}$. It follows, that the absolute reduction of the NRMSEs is higher for smaller sample size, in particular, if $N=27$ observations are used instead of $N=18$. Thus, in the situation under consideration, it is reasonable to consider at least $N=27$ observations.

Comparing the NRMSEs of the original design to the NRMSEs of the simultaneous *D*-optimal design (or the equidistant and *K*-means design, respectively), the following can be observed: The NRMSEs of the original design for $N=36$ are similar to the NRMSEs of the simultaneous *D*-optimal design for $N=27$. Similar observations are possible if the sample sizes are increased step wisely. That means that at least 9 more observations are necessary, if the original design is used, in order to achieve the precision of the simultaneous *D*-optimal design.

## Conclusion and outlook

This paper introduced two ways to construct designs that address the problem of the simultaneous inference of a large number of concentration–response relationships: the *K*-means design which is based on locally *D*-optimal designs of individual concentration–response curves and the *D*-optimal design for simultaneous inference which incorporates the distribution of the nonlinear parameters of the different concentration–response curves. In order to investigate the performance of these designs, we used the VPA-data set by Krug et al. [16] and constructed the corresponding designs for the relevant concentration–response relationships contained in this data set. Then the designs were compared to the design that was originally used for generating the VPA-data set and to an appropriate log-equidistant and an equidistant design. The comparison was done in terms of *D*-efficiencies and in terms of the performance for the simultaneous estimation of 15,233 concentration–response curves in a simulation study (imitating the original VPA-data).

In terms of *D*-efficiency and terms of the simulation study, we observed similar results: the simultaneous *D*-optimal design results in the most precise model fits for the different curves under consideration, the model fits obtained if the *K*-means and the equidistant design used are also appropriate. The log-equidistant design performs worse and the corresponding precision of the model fits is the lowest. Consequently, it is not recommendable to use the log-equidistant design if a large number of different concentration–response relationships (with considerably different shapes) should be estimated.

In general, it follows that the simultaneous *D*-optimal design improves the inference substantially. While the *K*-means design also performs well in the situation under consideration, it might be less feasible due to its construction. The construction of the simultaneous *D*-optimal design is straightforward by including a rough distribution of the nonlinear parameter values. Note that the equidistant design also performs well with respect to the considered measures in the situation under consideration, but it might perform worse in others. The advantage of the simultaneous *D*-optimal design is its flexibility: it can easily be adapted to the situation at hand, based on a distribution which can be predefined by the user.

The paper is based on the assumption that all concentration–response relationships are modelled appropriately by the same nonlinear regression function (with varying parameters). Different parametric regression functions could be assumed and the distribution of the occurrence of these curves could be included in the *D*-optimality criterion for simultaneous inference. Moreover, we restrict ourselves to the *D*-optimality criterion, other criteria could also be used to construct a design for simultaneous inference, e.g. addressing the precise estimation of the $\text{ EC}_{50}$ values or the prediction of responses at predefined concentrations. We leave the extension of these approaches to future research.

### Supplementary information

**Additional file 1.** Equivalence Theorem for $D$-optimal design for simultaneous inference, Additional Figures, Comparison of the designs grouped by parameter $\text{EC}_{50}$ and $h$.

## Data Availability

The data was originally provided by Krug et al. [16]. The data set as it is used in this publication can be found at https://github.com/schuermeyer/Designs-for-the-simultaneous-inference-of-concentration-response-curves. In addition the R-code and the main functions used in our analysis are provided.
