# Supplementary material for: Designs for the simultaneous inference of concentration–response curves
Source: BMC Bioinformatics. 2023 Oct 19;24:393. doi: 10.1186/s12859-023-05526-3 (PMC10588042; doi:10.1186/s12859-023-05526-3)
Supplement: Supplementary file 1 — Additional file 1. Equivalence Theorem for $D$D-optimal design for simultaneous inference, Additional Figures, Comparison of the designs grouped by parameter $\text{EC}_{50}$EC50 and $h$h. [file 12859_2023_5526_MOESM1_ESM.pdf]

Supplementary material

Leonie Schürmeyer, Kirsten Schorning and Jörg Rahnenführer

Equivalence Theorem for the D-optimal design for simultaneous inference

For the proof of statement (6) methods of convex analysis have to be used (see Silvey (1980) for details). As the criterion Ψ is concave due to the concavity of ψ_D, a design ξ^* maximizes Ψ if and only if its derivative in ξ^* in direction $(1-\alpha)\,\xi^* + \alpha\xi_x$ is smaller or equal to zero for all $x \in \mathcal{X}$. In particular, the derivative of the criterion $\Psi\,(\xi,\pi)$ evaluated in a design ξ in direction $(1-\alpha)\,\xi + \alpha\xi_x$ has to be calculated, where ξ_x is the design that puts weight "1" to an arbitrary but fixed point $x \in \mathcal{X}$.
In the situation of the criterion $\Psi(\xi,\pi)$ we consider the derivative:

$$\frac{\partial}{\partial\alpha}\Psi(\xi,\pi) = \lim_{\alpha\to 0}\frac{\Psi\left((1-\alpha)\xi + \alpha\xi_x\right) - \Psi\left(\xi,\pi\right)}{\alpha}$$

Applying classical rules of differentiation, this term can be rewritten by:

$$\frac{\partial}{\partial\alpha}\Psi(\xi,\pi) = \sum_{\theta\in\Theta}\frac{\pi(\theta)}{\psi_D\left(\xi^*,\theta\right)}\frac{\partial}{\partial\alpha}\psi_D\left(\xi,\theta\right) \tag{E1}$$

where $\frac{\partial}{\partial\alpha}\psi_D\left(\xi,\theta\right)$ is the derivative of the D-optimality criterion $\psi_D\left(\xi,\theta\right) = \left(\det\left(M(\xi,\theta)\right)\right)^{\frac{1}{p}}$ in ξ in direction $(1-\alpha)\xi + \alpha\xi_x$ it holds:

$$\begin{aligned}\frac{\partial}{\partial\alpha}\psi_D\left(\xi,\theta\right) =& \frac{1}{p}\left(\det(M(\xi,\theta))\right)^{\frac{1}{p}-1}\\ &\lim_{\alpha\to 0}\frac{\det\left(M((1-\alpha)\xi + \alpha\xi_x)\right) - \det\left(M(\xi,\theta)\right)}{\alpha}\\ =& \frac{1}{p}\left(\det(M(\xi,\theta))\right)^{\frac{1}{p}-1}\det\left(M(\xi,\theta)\right)\\ &\lim_{\alpha\to 0}\frac{\alpha\mathrm{tr}\left(M^{-1}(\xi,\theta)\left\{M(\xi_x,\theta) - M(\xi,\theta)\right\}\right)}{\alpha},\end{aligned} \tag{E2}$$

where the last equality follows by the fact that $\det(A+H) - \det(A) = \det(A)\mathrm{tr}(A^{-1}H)$, for matrices $A, H \in \mathbb{R}^{p\times p}$, for H small enough.

Rewriting $M(\xi_x, \theta) = \left(\frac{\partial}{\partial\theta}\eta(x,\theta)\right)\left(\frac{\partial}{\partial\theta}\eta(x,\theta)\right)^T$, (E2) can be reformulated by:

$$\begin{aligned}\frac{\partial}{\partial\alpha}\psi_D\left(\xi,\theta\right) =& \frac{1}{p}\left(\det\left(M(\xi,\theta)\right)^{\frac{1}{p}}\right)\\ &\left\{\left(\frac{\partial}{\partial\theta}\eta(x,\theta)\right)^T M^{-1}(\xi,\theta)\left(\frac{\partial}{\partial\theta}\eta(x,\theta)\right) - p\right\}\\ =& \frac{1}{p}(\det(M(\xi,\theta)))^{\frac{1}{p}} d(x,\xi,\theta),\end{aligned}$$

where $d(x,\xi,\theta)$ is given by the function depicted in (3). Inserting (E2) in formula (E1), we obtain $\frac{\partial}{\partial\alpha}\Psi(\xi,\pi) = s(x,\xi,\pi)$, where $s(x,\xi,\pi)$ is given in (6).

Additional Figures

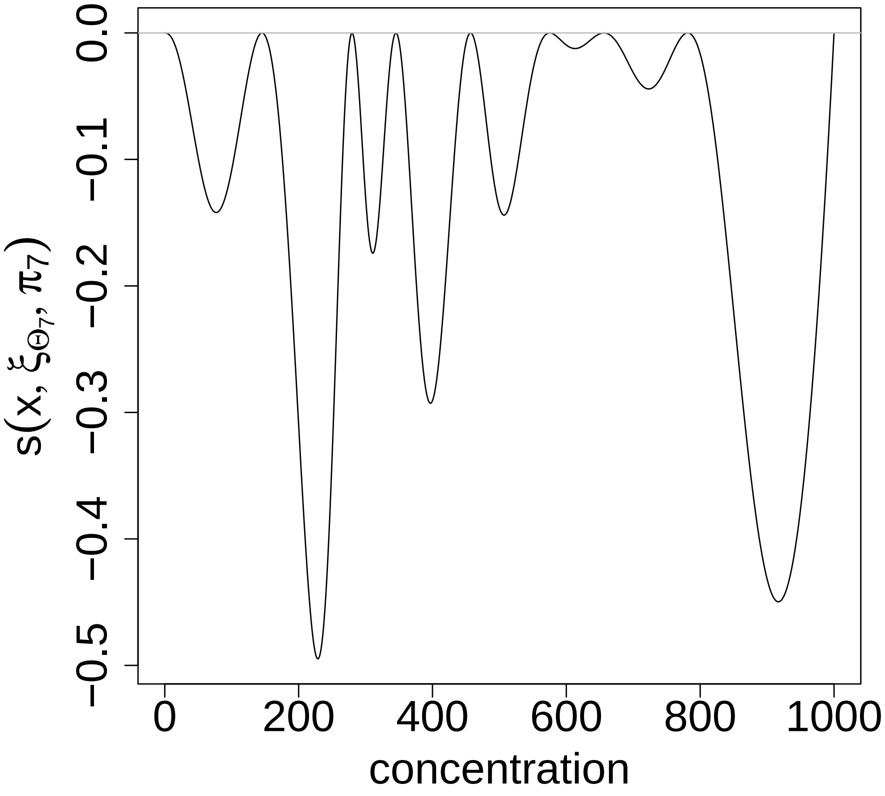

Figure S1: The plot shows the function $s(x, \xi_{\Theta_7}, \pi_7)$ in (6). Since the function is non-positive on the design space $\mathcal{X} = [0, 1000]$, the design ξ_{Θ_7} is D-optimal for simultaneous inference.

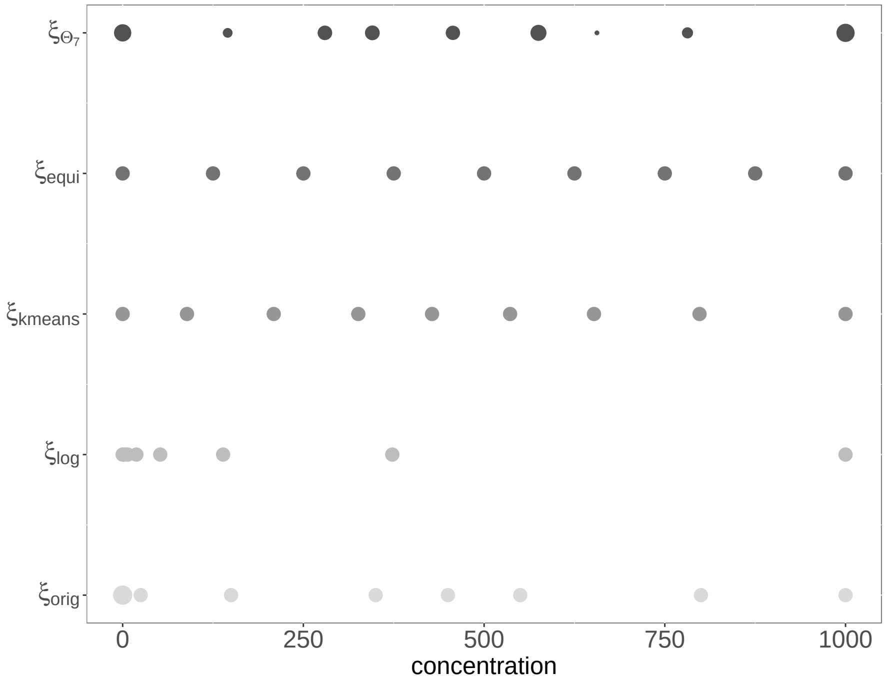

Figure S2: Support points of all considered designs with information of corresponding weights by size

Comparison of the different designs grouped by parameter EC_{50} and h

Comparison of the D-efficiencies grouped by parameter EC_{50} and h

In the left panel of Figure S3 the relative D-efficiencies grouped by design and EC_{50} are shown, where the outliers are removed for the sake of clarity. The corresponding plot including outliers can be found in Figure S4A. Note that we could not observe any structure in the outliers with respect to the design.

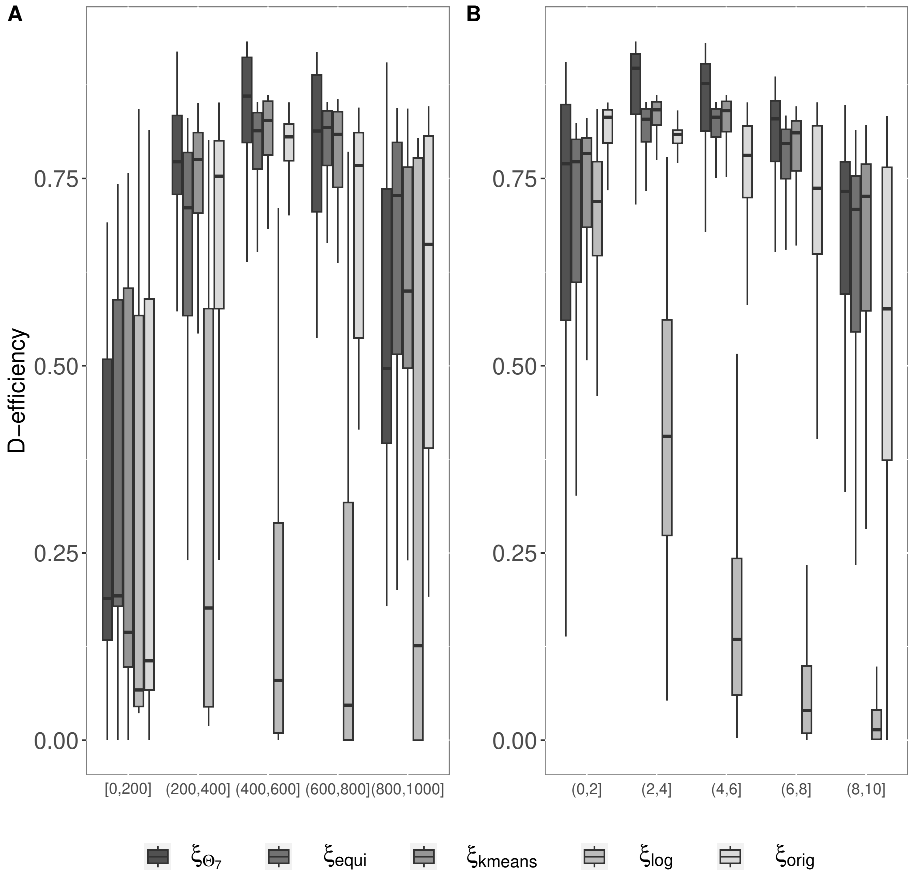

Figure S3: **A**: D-efficiency values for each gene regarding different designs divided by parameter h, **B**: D-efficiency values for each gene regarding different designs divided by parameter EC_{50}. The outliers are removed in both Figures.

The simultaneous D-optimal design leads to the greatest D-efficiencies for $\mathrm{EC}_{50} \in (200, 800]$. Note that for 84.5% of the 15 233 considered curves it holds $\mathrm{EC}_{50} \in (200, 800]$ and that the support of the used distribution π_7 only contains EC_{50} within $(200, 800]$, such that this effect is not surprising. For $\mathrm{EC}_{50} \leq 200$ (7.7% of all genes), the simultaneous D-optimal design still performs well compared to the other designs with respect to the medium D-efficiencies. Considering the complete distribution of D-efficiencies the equidistant design results in the best D-efficiencies, although the D-efficiencies are small for all considered designs if $\mathrm{EC}_{50} \leq 200$. If $\mathrm{EC}_{50} > 800$ (7.8% of all genes), the simultaneous D-optimal design and the log-equidistant design result in the smallest D-efficiencies. In this case, the equidistant design and the original designs perform best, as these designs contain two support points that are close to the assumed EC_{50}, which are $875, 1000$ and $800, 1000$, respectively.

In the right panel of Figure S3 the relative D-efficiencies grouped by design and the steepness parameter h are shown, where the outliers are also removed for the sake of clarity. The corresponding plot including outliers can be found in Figure S4B. Note that we could not observe any structure in the outliers with respect to the design. For all groups with $h > 2$ (87.9% of all genes), the simultaneous D-optimal design leads to the greatest D-efficiencies compared to the other designs, both with respect to the medians and the lower quartiles. The original design's D-efficiencies are substantially smaller for $h > 2$, whereas the equidistant and the K-means design have similar D-efficiencies that lie between the ones of the original and the simultaneous D-optimal design. For $h \leq 2$ (12.1% of genes), the original design results in the highest D-efficiencies. In particular, the lower quartile of the D-efficiencies of the original design is greater than the upper quartiles of the D-efficiencies of the equidistant, log-equidistant and the K-means design. The D-efficiencies of the simultaneous D-optimal design are most varying, with minimal value 0.07 and highest D-efficiency 0.91 for $h \leq 2$. This effect is explained by considering the discrete distribution π_7 that is used for the construction of ξ^*_θ: $\pi_7(\theta) = 0$ for all $\theta = (\mathrm{EC}_{50}, h)$ with $h \leq 2$, which means, it does not aim for high D-efficiencies for $h \leq 2$.

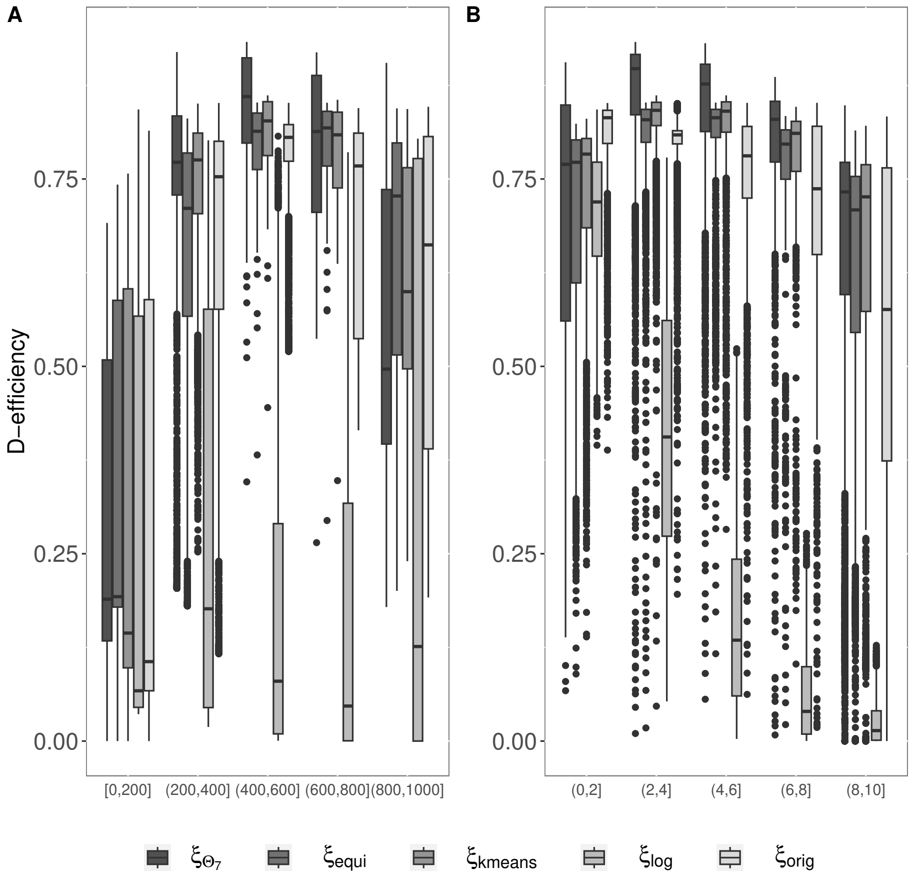

Figure S4: **A**: D-efficiency values for each gene regarding different designs divided by parameter h, **B**: D-efficiency values for each gene regarding different designs divided by parameter EC_{50}

Simulation results grouped by parameter EC_{50} and h

In the left part of Figure S5 the NRMSEs are shown grouped by EC_{50} and design, where the outliers are removed for the sake of clarity. The corresponding plot including outliers can be found in Figure S6A. Note that we could not observe any structure in the outliers with respect to the design.

If $\mathrm{EC}_{50} \in [200, 600)$, the box plots of the NRMSEs are similar to the ones of the general analysis for all designs under consideration. In particular, the simultaneous D-optimal design and the K-means still perform best, whereas the NRMSEs of the equidistant design are slightly larger. If $\mathrm{EC}_{50} \in [600, 1000)$, the NRMSE based on the equidistant design are the smallest compared to the other designs. Note that we observed a similar behaviour of the D-efficiencies of

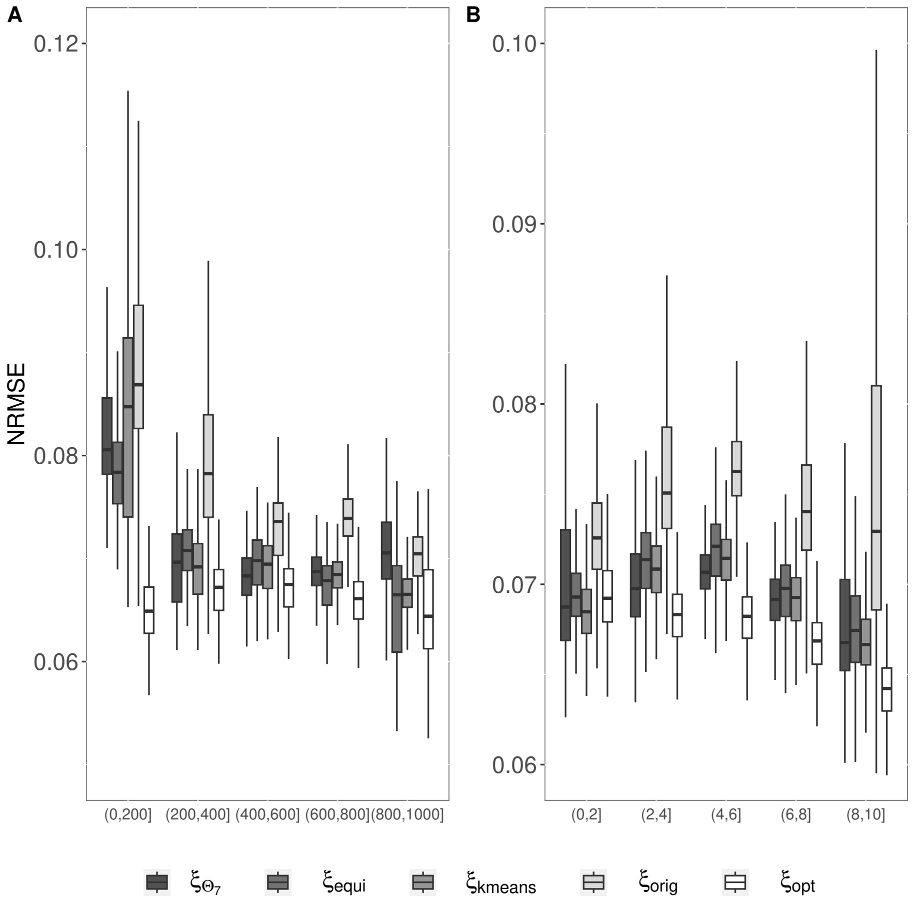

Figure S5: **A**: NRMSE values for each gene regarding different designs with 27 measurements and grouped by parameter EC_{50}, **B**: NRMSE values for each gene regarding different designs with 27 measurements and grouped by parameter h. The outliers are removed in both Figures.

the equidistant design for that case. For small EC_{50}-values, that is $EC_{50} \leq 200$, the considered designs result in higher NRMSE in general, whereas the ranking of the NRMSEs with respect to the designs stays almost the same. In particular, the equidistant, the K-means and the simultaneous D-optimal design still have similar NRMSEs, whereas the equidistant slightly outperforms the others. The original design performs worse with respect to the NRMSE independent from the restriction on the EC_{50}-value.

In the right part of Figure S5, the NRMSEs are shown grouped by h and the design, where the outliers are removed for the sake of clarity. The corresponding

plot including outliers can be found in Figure S6B. Note that we could not observe any structure in the outliers with respect to the design. As for the EC_{50}, the NRMSEs of the different designs are only slightly varying if considered restricted to h. In particular, the simultaneous D-optimal design results in higher varying NRMSEs with a median equal to 0.069 and 0.75%-quantile at 0.073, for $h \leq 2$. Note that we observed a similar effect for the corresponding D-efficiencies, which could be explained by the fact that values $h \leq 2$ were not considered for the construction of this design. If $h \in (8, 10]$, the original design results in higher varying NRMSEs. Note that this effect was also observed for the corresponding D-efficiencies of that design (see Figure S3.B).

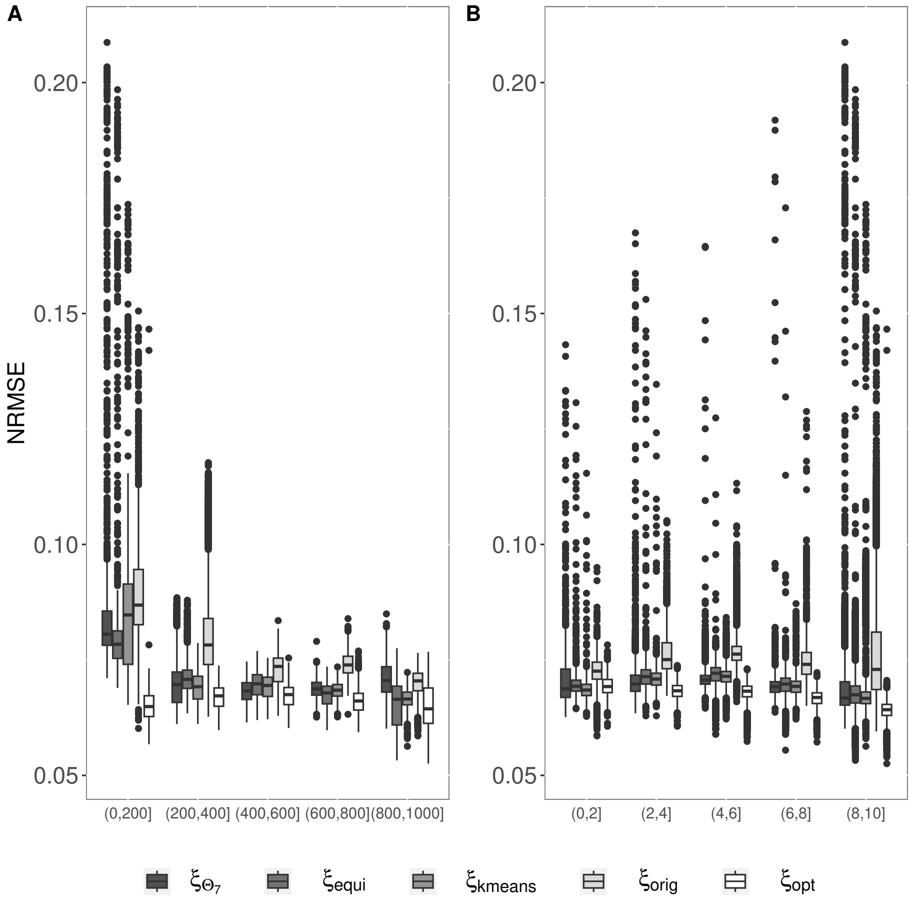

Figure S6: **A**: NRMSE values for each gene regarding different designs with 27 measurements and grouped by parameter EC_{50}, **B**: NRMSE values for each gene regarding different designs with 27 measurements and grouped by parameter h
